# The impact of intelligent robot service failures on customer responses --a perspective based on mind perception theory

**DOI:** 10.3389/frobt.2025.1581083

**Published:** 2025-06-30

**Authors:** Mengting Gong, Aimei Li, Junwei Zhang

**Affiliations:** ^1^ School of Public Administration, Guangdong Mechanical and Electrical Polytechnic, Guangzhou, China; ^2^ School of Management, Jinan University, Guangzhou, Guangdong, China; ^3^ Guangdong Post and Telecommunications Talent Service Co., Ltd., Guangzhou, China

**Keywords:** intelligent robot service failure, mind perception theory, agency, experience, customer response

## Abstract

As intelligent robots are widely applied in people’s work and daily life, intelligent robot service failures have drawn more attention from academics and practitioners. Under the scenarios of intelligent robot service failures, most existing studies focus on service providers’ remedies for the failures and customers’ psychological responses to such failures. However, few have systematically explored the impacts of intelligent robot service failures on customers and their internal psychological mechanisms. This paper adopts the framework of mind perception theory to systematically categorize the types of intelligent robot service failures and explores their impact on customer responses from the dimensions of agency and experience. By constructing a theoretical framework to analyze the effects of intelligent robot services on customers, it provides valuable theoretical insights for scholars in the field of intelligent marketing and sheds light on the psychological mechanisms of customers under intelligent robot service failure scenarios.

## 1 Introduction

According to statistics from the China Commercial Industry Research Institute, the cumulative output of service robots in China reached 9.2144 million units in 2021, with the market size projected to hit 52.43 billion yuan by the end of 2022. Compared to human employees, service robots possess numerous advantages, including enhanced service efficiency, economies of scale, and reduced operational costs (Liu et al., 2023). The increase in the number of units indicates that Chinese consumers’ demand for service robots has increased sharply, and the market penetration rate of service robots continues to rise. However, constrained by the current levels of artificial intelligence (AI), the diversity of service scenarios, and enterprise application costs, intelligent robots inevitably encounter service failures during service delivery (Jiang et al., 2023),Intelligent robot service failure refers to situations where customers’ service requests are either rejected by the robots (Yu et al., 2024) or the robots fail to perform specific tasks assigned by customers (Longoni and Cian, 2022). Recent years have witnessed nearly 100,000 complaints related to intelligent robot service failures on complaint platforms. Most customers complain about robots being “inhumane” or “incapable of understanding and communicating with them.” Additionally, despite over a decade of development, robot restaurants, whether from smaller brands or industry giants such as Country Garden’s Qianxi Robot or Alibaba’s Hema Robot. Hema Robot, has faced numerous challenges, with many initiatives ending without success. Such instances of intelligent robot service failures not only diminish customer satisfaction, purchase intentions, and positive evaluations of companies (Crolic et al., 2022), but also these types of failures heighten resistance toward products or services offered by intelligent robots (Wien and Peluso, 2021). Service failures undermine consumers’ expectations of brand value (Srinivasan and Sarial-Abi, 2021), and infringe upon their rights to know (Xu, 2021) and privacy (Leo and Huh, 2020).

Under the scenarios of intelligent robot service failures, existing studies primarily focus on service providers’ remedies for the failures and customers’ psychological responses to such failures. Studies on the former include the two main dimensions of material and psychological remedies offered by service providers (Lv X. et al., 2021). For example, there are two typical service compensation methods in the interaction between customers and robots, namely, functional compensation and symbolic compensation (Jiangang et al., 2022). Azemi et al. (2019) proposed strategies for recovering from intelligent service failures, such as psychological apologies, financial compensation or discounts, and downward social comparisons (i.e., comparisons with less fortunate customers). These studies provide valuable insights for service providers in addressing robot service failures. Customers’ psychological reactions to intelligent robot service failures have also garnered significant academic attention. For instance, when customers feel angry after a robot service failure, they exhibit dissatisfaction and reduced purchase intentions toward anthropomorphized chatbots (Crolic et al., 2022). Similarly, when chatbots fail to recognize context, customers’ intention to use them diminishes (Sheehan et al., 2020). Furthermore, consumers are less trusting and reliant on intelligent algorithms for handling tasks perceived as inherently subjective (Castelo et al., 2019). Thus, existing research in the field of intelligent robot service failures has achieved considerable progress in understanding service providers’ remedies for service failures and customers’ responses to such failures.

Despite these findings, existing studies on intelligent robot service failures lack theoretical integration, hindering our understanding of customers’ psychological mechanisms in such failure scenarios. Thus, this paper aims to review the existing literature on intelligent robot service failures from the perspective of mind perception theory, establishing a more unified and systematic theoretical framework to elucidate the impact of such failures on customer cognition, attitudes, and behaviors. Databases searched included China National Knowledge Infrastructure (CNKI), Google Scholar, and some former interview data. Specifically, the CNIK is an academic platform founded in June 1999 by Tsinghua Univeristy, which involved many professional resources and services, including academic journal library, outstanding doctoral and master’s degree theses, published books, important conference papers, encyclopedias, yearbooks and government documents. Moreover, Google scholar is a free web search engine for academic articles, its’ index covers most of the world’s published academic journals, books, conference minutes, patent reports, technical and experimental reports. In addition, as the lead author, I have conducted several research interviews with some customers in Guangdong, mainland China, and collated relevant materials as primary data. According to these resources above, the main search terms were types of intelligent robot service failure, mind perception theory, agency, experience, and customer responses.

The paper first analyzes technical service failures, focusing on internal faults in the robot’s technical components or mechanisms, including hardware and software failures. These technical service failures lead to functional service failures by intelligent robots. The paper then discusses functional service failures of intelligent robots, wherein robots fail to deliver the expected or requested services, including service request failures and normative compliance failures. Functional service failures may, in turn, contribute to technical service failures. To facilitate analysis, the paper categorizes intelligent robot service failures into two main types: technical and functional service failures. Based on these categories, the paper further explores customers’ psychological mechanisms under different service failure scenarios. Subsequently, the paper proposes moderation mechanisms from both the robot and customer levels, covering factors such as the severity of service failures, communication styles, artificial intelligence anthropomorphism, social classes, and interpersonal interaction needs. Finally, the paper elaborates on the impact of intelligent robot service failures on customers’ cognition, attitudes, and behaviors.

## 2 Classification of intelligent robot service failures

Some of the existing literature indicate that intelligent robot service failure is a degraded state in which the behavior or service executed by the robot system deviates from its ideal, normal, or correct functionality. Intelligent service failures include perceivable failures and actual failures caused by robots executing actions correctly as programmed (Liu et al., 2023). Intelligent robot service failure, as a perspective failure, occurs when a customer’s service request is rejected by the intelligent robot (Yu et al., 2024). When algorithmic decisions are made that are beneficial to the consumer themselves, theses decisions sometimes elicit more negative reactions, resulting in algorithmic service failures by intelligent robots (Yalcin et al., 2022). When intelligent robots recommend hedonic products or services that customers perceive as mismatched with their unique preferences, intelligent robot service failures can be the result (Longoni and Cian, 2022).

Actual failures in intelligent robot service failures are quite common in daily consumption scenarios. For example, an intelligent voice fax server and interactive voice response system (IVR) of a telecom company can efficiently provide customers with technologies and functions such as information inquiries, transaction services, business handling, complaint feedback, and market surveys. However, when faced with more complex real-world situations, these systems often encounter technical and functional service failures. Regarding the former, technical service failures occur when the intelligent voice fax server malfunctions during data transmission, leading to data loss, incorrect data, or timing errors, resulting in communication service failures (Brooks, 2017). Additionally, when the intelligent voice fax server encounters anomalies or segmentation faults while processing files, processing service failures occur (Brooks, 2017). As for functional service failures, when the services provided by the IVR system fail to support consumers in completing their transactions or achieving their goals, system service failures occur (Tan et al., 2016). Furthermore, when the IVR system fails to protect customer privacy and rights or violates assumptions of basic code of ethics during the execution of related tasks, these may lead to interaction failures (Giuliani et al., 2015). Thus, intelligent robot service failures can primarily be further categorized into technical and functional service failures.

### 2.1 Technical service failures

#### 2.1.1 Hardware service failures

Lu et al. (2020) noted that technical failures are malfunctions caused by technological defects in robots. Here, the focus is on internal technical components or mechanisms of the robot system, including hardware and software service failures. Hardware service failures include physical failures and communication failures. Physical failures refer to failures caused by physical errors in effectors, sensors, control systems, power supplies, or communication systems (Honig and Oron-Gilad, 2018). Communication failures, as proposed by Brooks (2017), relate to data directly transmitted between modules, including lost data (incomplete messages or discarded packets), incorrect data (data generated during transmission), timing errors (data sent too early or received too late), and extra data (sending more information than expected). Hardware failures in robot services refer to malfunctions or damage to the robot’s physical components or hardware devices, preventing the robot from functioning normally or performing expected tasks. Hardware failures may involve various components, such as sensors, actuators, power supplies, and circuit boards. When these hardware components encounter problems, robots may exhibit unstable behavior, fail to respond to commands, or completely cease functioning.

#### 2.1.2 Software service failures

Software service failures are further categorized into design failures and processing failures (Honig and Oron-Gilad, 2018). Design failures in software systems refer to user interface design flaws that disrupt users’ attention (Lu et al., 2020). For instance, some intelligent robots fail to use concealed designs or scenario paging in their interactive interfaces, reducing the likelihood of effective user-robot interactions. Processing failures refer to unhandled exceptions, segmentation faults, or loss events, such as logical or semantic errors and unforeseen situations (Brooks, 2017). Design failures in intelligent robot software systems are not only reflected in prolonged interface loading time but also in information overload, making it difficult for consumers to focus on the services they need. Moreover, data processing failures often lead to incorrect interpretations of perceived data by intelligent robots, resulting in negative psychological attitudes and decision-making behaviors from customers.

### 2.2 Functional service failures

#### 2.2.1 Service request failures

Outcome failures refer to situations where the fundamental services expected by customers are not fulfilled. Such failures are closely tied to the explicit promises made by service providers and the core results of the service (Peng and Wang, 2007). When intelligent robots lack the capability to deliver core services as expected by consumers, service outcome failures often occur (Li et al., 2019). For example, the world’s first hotel operated by service robots opened in Japan in 2015. However, when the luggage-carrying robots were deployed, customers discovered that these robots could not climb stairs or venture outside. The customers thought these robots could not provide core services as expected, resulting in service request failures.

Tan et al. (2016) proposed that system failures occur when the services provided by Artificial Intelligence (AI) fail to facilitate consumers in completing their transactions or achieving their goals. For system failures, their quality dimensions are associated with response time and reliability, often manifesting as delayed or inappropriate responses when using AI assistants (Sun et al., 2022). When intelligent robot systems malfunction, they fail to deliver effective services to consumers, making it difficult for customers to obtain the services they need. Moreover, system failures may manifest as system crashes, non-responsiveness, or slow operation. For example, when an Ecovacs robot equipped with AI visual recognition technology (including sound source localization, visual recognition, and precise navigation) and intelligent obstacle avoidance fails to start, with red lights flashing, users perceive it as failing to meet their expectations and service needs, leading to service request failures (Tarafdar and Bose, 2021).

#### 2.2.2 Normative compliance failures

Honig and Oron-Gilad (2018) discussed interaction failures as issues arising from uncertainties in interactions with the environment, other agents, and humans, such as violations of social norms. Further, violating social norms refers to intelligent robots failing to adhere to basic code of ethics and failing to protect customer privacy and rights (Giuliani et al., 2015). For instance, humanoid robots bring about various legal and ethical risks, most notably the risks of infringement of portrait rights, advertising endorsement liabilities, and risks associated with the “uncanny valley effect.” More specifically, Crolic et al. (2022) clarified that the “uncanny valley” means the tendency for a robot to elicit negative emotional reactions when it closely resembles a human because robots do not perform in the agentic manner that their human resemblance would imply. In other words, the robots’ behavior violates the expectations elicited by their highly anthropomorphic facade. These risk factors affect the likelihood and effectiveness of the interactions between robots and customers.

On the other hand, there are human failures. Human failures refer to errors caused by human actions, including those due to memory or attention lapses (e.g., forgetting to turn off a robot) or deliberate misconduct, such as intentionally guiding a robot to crash into obstacles (Honig and Oron-Gilad, 2018). Excessive control over automated systems by humans can result in fatal mistakes. Advocates of autonomous vehicles, for example, highlight how deliberate human errors can lead to accidents (Shneiderman, 2020). When using intelligent robots, human employees often cause service failures due to their limited knowledge and skills. Therefore, based on the above literature on service failure types, this paper categorizes intelligent robot service failures into two main categories: technical and functional failures (see Table 1).

**TABLE 1:** Classification of intelligent robot service failures.

Author	Year	Theory	Definition of service failures	Classification of service failures
Honig and Oron-Gilad (2018)	2018	Information processing theory	Physical failures: failures caused by physical errors in effectors, sensors, control systems, or power supplies	Technical service failures
Brooks (2017)	2017	Media richness theory	Communication failures: failures caused in direct data transmission, such as lost data, incorrect data, and timing errors	Technical service failures
Lu et al. (2020)	2020	Cultural dimension theory	Design failures: user interface design flaws that disrupt users’ attention	Technical service failures
Brooks (2017)	2017	Media richness theory	Processing failures: unhandled exceptions, segmentation faults, or loss events, such as logical or semantic errors	Technical service failures
Peng and Wang (2007)	2007	Self-threat theory	Outcome failures: failures resulting from providers not fulfilling the basic services expected by customers	Functional service failures
Tan et al. (2016)	2016	Expectancy disconfirmation theory	System failures: Services provided by AI fail to facilitate consumers in completing their transactions or achieving their goals	Functional service failures
Giuliani et al. (2015)	2015	Signal theory	Interaction failures: intelligent robots fail to adhere to basic code of ethics and fail to protect customer privacy and rights	Functional service failures
Honig and Oron-Gilad (2018)	2018	Information processing theory	Human failures: mistakes caused by humans, including memory or attention lapses	Functional service failures

## 3 Mind perception theory

Based on Mind Perception Theory proposed by social psychologist Kurt Gray in 2007, this paper explores how individuals interpret and predict through their mental processes (Gray and Wegner, 2012). Mind Perception Theory explains how individuals interpret the mental states of others through observed information or phenomena (Uysal et al., 2022), primarily by perceiving others’ thoughts along two dimensions: agency and experience. The agency dimension of mind perception encompasses the capacity to plan and act-having self-control, judgment, planning, thinking, and action (Srinivasan and Sarial-Abi, 2021). The experience dimension refers to the capacity to sense and feel, which relates to encompassing emotional and physical sensations such as hunger, fear, pain, anger, and joy (Srinivasan and Sarial-Abi, 2021).

When intelligent robot service failures occur, customers experience different forms of service failures (i.e., technical and functional service failures). They assess the robot’s agency and experience based on the observed or perceived failure information. The agency dimension includes intentions, inference, goal pursuit, and memory outcomes (Shank et al., 2019), while the experience dimension focuses on emotional aspects, including fear, pain, joy, and anger (Appel et al., 2020). The agency dimension also emphasizes capacity. Failures attributed to robots lacking capacity are considered uncontrollable, whereas those linked to insufficient effort are deemed controllable (Vander Woerdt and Haselager, 2019). The experience dimension focuses more on emotional states, such as pain, personality, and emotion (Sullivan and Fosso, 2022). For instance, customers often perceive AI recommendations for hedonic products as inferior to human suggestions, undermining their experiential perception and amplifying their aversion to such AI-driven recommendations (Wien and Peluso, 2021). Furthermore, Yam et al. (2021) explored from the agency dimension of mind perception how stronger perceived agency intensifies the adverse effects of service failures on customer satisfaction when faced with intelligent robot service failures. And Kim et al. (2023) highlighted from the experience dimension that intelligent robots’ lack of certain human qualities (e.g., feelings) leads to unethical customer behaviors, such as lying, cheating, and even theft.

Customers’ different perceptions of intelligent robot service failures continuously influence their responses to such failures, including their cognitive processes, consumption attitudes, and decision-making behaviors. Thus, mind perception theory not only elucidates how information is shared, decisions are made, and actions are coordinated between agents (humans and robots) but also reveals internal mental processes and human-robot interaction mechanisms. Mind perception theory provides vital theoretical support for effective human-robot collaboration. Undoubtedly, the applicability of mind perception theory in the field of intelligent robot service failures should expand.

## 4 Exploration of mediating mechanisms in intelligent robot service failures

### 4.1 Perceived agency and experience brought by technical service failures

Technical service failures are malfunctions caused by technological defects in robots, focusing on internal technical components or mechanisms of the robot system, including hardware and software service failures (Lu et al., 2020). Hardware service failures include physical failures and communication failures. Physical failures refer to failures caused by physical errors in effectors, sensors, control systems, power supplies, or communication systems (Honig and Oron-Gilad, 2018). Then, Brooks (2017) proposed that communication failures are related to data directly transmitted between modules, including lost data, incorrect data, timing errors, and extra data. Software service failures are primarily categorized into design failures and processing failures (Honig and Oron-Gilad, 2018). Design failures refer to user interface design flaws that disrupt users’ attention (Lu et al., 2020) while processing failures are defined as unhandled exceptions, segmentation faults, or loss events, such as logical errors (Honig and Oron-Gilad, 2018). Additionally, Zhang (2021) pointed out that in human-robot interactions, logical and semantic errors exhibited by intelligent robots stem from their lack of capability, which constitutes a violation of expected behavior.

Additionally, when intelligent robots experience the aforementioned technical service failures, customers perceive the robots’ agency and experience through the observed or received information or phenomenon about the service failures. First, customers’ perceptions of the agency of intelligent robots are reflected in three aspects: 1) Problem-solving Ability, 2) Service Innovation Ability, and 3) Environmental Adaptability. Problem Solving Ability primarily corresponds to a sense of control (Jiangang et al., 2022). When technical service failures occur, customers perceive a lower sense of control from the intelligent robot, as it fails to achieve the expected results in a given environment. Service Innovation Ability refers to the consumers’ perceptions of intelligent innovation, including creative novelty, technological novelty, and relative advantages (Lv X. et al., 2021). Customers tend to perceive a weaker service innovation ability of the intelligent robot when technical service failures occur. Environmental Adaptability mainly refers to flexibility. Intelligent robots can not only fulfill personalized demands of consumers but also raise consumer expectations for their flexibility, thereby enhancing customer satisfaction (Yu et al., 2024). However, when a robot refuses a customer’s service request, it is often perceived as lacking flexibility, which makes the customers have negative attitudes toward its services (Yu et al., 2024). Thus, technical failures result in customers perceiving lower levels of agency in intelligent robots.

Next, customers’ perceptions of the experience dimension during technical service failures focus on enthusiasm and guilt. Roy and Naidoo (2021) highlighted that robots adopting human-like conversational styles exhibit both enthusiasm (perceived friendliness and trust) and competence (intelligence and skills). Furthermore, customers who focus on immediate rewards or short-term benefits prefer interacting with enthusiastic rather than competent robots, leading to favorable product decisions (Roy and Naidoo, 2021). However, when humanoid robots lack enthusiasm, they elicit more negative attitudes and behaviors from customers compared to non-humanoid robots (Song and Kim, 2022). Guilt is an unpleasant moral emotion. When robot avatars make offensive statements, participants experience guilt over the robot’s offensive actions, even if they were not directly responsible for the robot’s misconduct (Aymerich-Franch et al., 2020). This research emphasizes the importance of moral considerations and legal issues related to robot technology (Aymerich-Franch et al., 2020). Therefore, when intelligent robot technical service failures occur, customers will reduce their positive experiential perceptions and increase their negative experiential perceptions of robots. This demonstrates the strong link between technical service failures of intelligent robots and the agency and experience dimensions in mind perception theory.

### 4.2 Perceived agency and experience brought by functional service failures

Functional service failures focus on the robot’s inability to deliver the expected or requested functions and services, including service request failures and normative compliance failures. Service request failures include outcome failures and system failures, while normative compliance failures include interaction failures and human failures. Outcome failures refer to failures resulting from robots not fulfilling the basic services expected by customers. When intelligent robots lack the capability to deliver core services as expected by consumers, service outcome failures often occur (Li et al., 2019). Tan et al. (2016) proposed that system failures occur when the services provided by AI fail to facilitate customers in completing their transactions or achieving their goals. On the other hand, normative compliance failures include interaction failures and human failures. Giuliani et al. (2015) described interaction failures as situations where intelligent robots fail to adhere to basic code of ethics and fail to protect customer privacy and rights. Besides, human failures refer to errors caused by human actions, including those due to memory or attention lapses or deliberate misconduct (Honig and Oron-Gilad, 2018).

Specifically, when intelligent robots experience functional service failures, customers perceive the robots’ agency and experience through the observed or received information or phenomenon about the service failures. Customers’ perceptions of the agency of intelligent robots are primarily reflected in three aspects: problem-solving ability, service innovation ability, and environmental adaptability.

**FIGURE 1:**
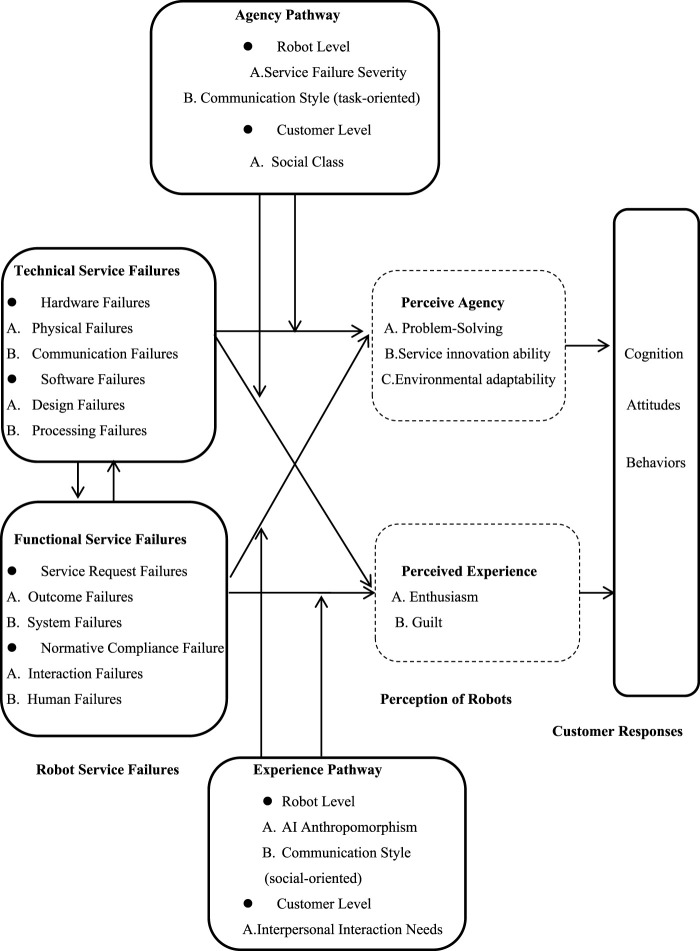
Theoretical framework of mediating mechanisms in intelligent robot service failures.

Firstly, problem-solving ability primarily corresponds to controllability. Controllability refers to the degree to which the service provider could avoid system failure. The robot’s controllability to service failures depends on its capability (Hou, 2021). When the functional service failures of an intelligent robot occur, its perceived controllability will also be reduced because it is unable to prevent failures. Secondly, service innovation ability refers to an individual’s perception ability of the technology or system (Um et al., 2020) When functional service failures of intelligent robots occur, customers lower their perception of intelligent technologies or systems. Environmental adaptability mainly refers to flexibility. Lv X.Y. et al. (2021) mentioned that unlike trained professionals, AI assistants in service industries learn from received data and execute tasks according to pre-set programs. However, their limited ability to adapt flexibly to problems increases the likelihood of functional service failures. Thus, functional service failures of intelligent robots do weaken customers’ perceptions of their agency. In addition, customers’ experience perceptions of intelligent robot functional service failures mainly focus on enthusiasm and guilt, the specific research framework is shown in Figure 1.

## 5 Exploration of moderating mechanisms for customer responses to intelligent robot service failures

### 5.1 Agency moderation pathway

When intelligent robots experience technical service failures, the agency pathway can be analyzed at both the robot and customer levels. In the first place, at the robot level, Jiang et al. (2023) defined service failure severity as the degree of loss perceived or assessed by customers following a service failure. As service failure severity increases from minor errors to serious accidents, the information individual concerns about also differs (Jiang et al., 2023). The greater the perceived loss, the more severe the service failure. Conversely, in less severe service failure scenarios, customers experiencing lower losses are less motivated to process related information and are more likely to perceive the robot’s innovation potential (Jiang et al., 2023). Thus, the severity of service failures moderates the impact of intelligent robot technical service failures on customers’ perceptions of the robot’s agency abilities. Moreover, the communication styles of intelligent robots can be divided into two categories: task-oriented and social-oriented. The task-oriented communication style emphasizes AI achieving specific goals, organizing conversations purposefully, improving task efficiency, and minimizing communication costs (Wang et al., 2021). Liu et al. (2023) noted that after a service failure, a task-oriented communication style helps customers perceive the robot’s professionalism and capabilities, fostering cognitive trust based on responsibility and reliability. Therefore, at the robot level, both the task-oriented communication style and service failure severity moderate the impact of intelligent robot technical service failures on customers’ perceptions of the robot’s agency abilities, which subsequently influences customers’ cognitive, attitudinal, and behavioral responses to robot service failures (Zhang and Wang, 2022).

Next, from the perspective of social class at customer level, in credit-based service settings, high social-class consumers are more inclined to choose services provided by robots compared to lower social-class consumers (Yao et al., 2022). Furthermore, customers from lower social classes perceive highly anthropomorphic robots as less intelligent, which diminishes their willingness to use them. In contrast, higher social-class customers perceive highly anthropomorphic robots as more intelligent, enhancing their willingness to use them (Zhang and Wang, 2022). Thus, social class moderates the impact of technical service failures on customers’ experience perceptions (including enthusiasm and guilt), resulting in varied customer responses to intelligent robot service failures.

### 5.2 Experience moderation pathway

When intelligent robots experience functional service failures, the experience pathway can be analyzed at both the robot and customer levels. On the robot level, when service functional failures involve interaction failures, such as failing to adhere to basic code of ethics or failing to protect customers’ privacy and rights (Giuliani et al., 2015) various legal and ethical risks may arise, including the risks of infringement of portrait rights and risks associated with the “uncanny valley effect.” For instance the “uncanny valley effect” means a person’s response to a humanlike robot will abruptly shift from empathy and acceptance to revulsion as the robot approaches a lifelike appearance but fails to attain it (Han et al., 2023). Therefore, enterprise managers should regulate the degree of anthropomorphism in AI systems to maintain an optimal level (Kim et al., 2019). By reducing excessive human-like characteristics, enterprise managers can enhance consumers’ perceptions of the robot’s controllability, thus facilitating more effective service delivery (Jiangang et al., 2022) and promoting more satisfactory consumer decisions. Additionally, the social-oriented communication style of intelligent robots plays a role. When intelligent robots fail to deliver core services as expected by customers, resulting in outcome failures (Li et al., 2019), a social-oriented communication style fosters personalized interactions with customers, strengthens the psychological connection between the robot and customer, and raises customer expectations of the robot’s flexibility. This, in turn, increases customer satisfaction with the robot’s functional services (Yu et al., 2024) and leads to more effective decision-making responses, including their cognitive processes, consumption attitudes, and decision behaviors. In such scenarios, AI anthropomorphism and a social-oriented communication style moderate the impact of functional service failures on customers’ perceptions of the robot’s agency abilities (e.g., problem-solving ability and environmental adaptability), thereby influencing customers’ cognitive, attitudinal, and behavioral responses to intelligent robot functional service failures.

**FIGURE 2:**
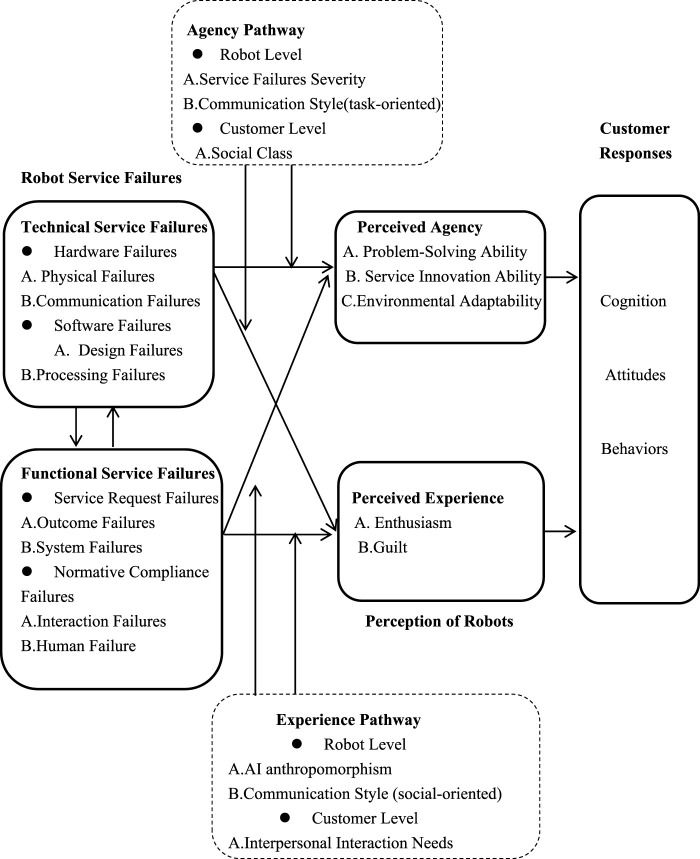
Theoretical framework of moderating mechanisms for customer responses to intelligent robot service failures.

At the customer level, the higher their need for interpersonal interaction, the stronger their intention to adopt anthropomorphized chatbots (Sheehan et al., 2020) thereby enhancing their experience perceptions of intelligent robots, including enthusiasm and guilt. Thus, customers’ interpersonal interactions moderate the impact of functional service failures on customers’ experience dimensions, resulting in varied customer responses to intelligent robot service failures. The specific research framework is shown in Figure 2.

## 6 General discussion

With the continuous development and implementation of intelligent technologies such as voice recognition, machine learning, autonomous navigation and positioning, intelligent robots have become the darlings of the market across various fields, showing rapid growth momentum. Accordingly, this paper provided synthesis and summarized the existing literature, revealing that customers encounter different types of failures when intelligent robots fail to deliver services. The paper first explained technical service failures, focusing on internal faults in the robot’s technical components or mechanisms, including hardware and software failures. Such service failures often lead to functional service failures of intelligent robots. The paper thus discusses functional service failures of intelligent robots, wherein robots fail to deliver the expected or requested services, including service request failures and normative compliance failures. Similarly, functional service failures may, in turn, contribute to technical service failures. Thus, the paper categorized intelligent robot service failures into two main types: technical and functional service failures. When intelligent robots experience technical or functional service failures, it impacts customers’ perceptions of the robots’ agency (problem-solving ability, service innovation ability, and environmental adaptability) and experience (enthusiasm and guilt). Subsequently, the paper proposes corresponding moderation mechanisms from both the robot and customer levels, covering factors such as the severity of service failures, communication styles, AI anthropomorphism, social classes, and interpersonal interaction needs. These mechanisms lead to different customer responses to intelligent robot service failures, including cognition, attitudes, and behaviors. Finally, the discussion now turns to the theoretical significance, practical implications, research limitations, and future research directions.

### 6.1 Theoretical significance

The theoretical significance of this study is mainly reflected in two aspects. First, unlike existing theories in the intelligent marketing field, such as media richness theory (Brooks, 2017), expectancy disconfirmation theory (Tan et al., 2016) and cultural dimension theory (Lu et al., 2020), this paper applies Mind Perception Theory as proposed by social psychologist Kurt Gray as it relates to using the two dimensions of perceived agency and perceived experience as mediating mechanisms between intelligent robot service failures and customer responses to these failures. From the perspective of perceived agency, dimensions include problem-solving ability, service innovation ability, and environmental adaptability, while from the perspective of perceived experience, dimensions mainly include enthusiasm and guilt. Second, this paper uses the two dimensions of agency and experience under Mind Perception Theory as pathways to moderate the relationship between intelligent robot service failures and customer perceptions of robots. Each pathway is analyzed from both the robot and customer levels, including aspects such as service failure severity, communication styles, AI anthropomorphism, social classes, and interpersonal interaction needs. This ultimately leads to varied customer responses to intelligent robot service failures (including cognition, attitudes and behaviors). Indeed, the theoretical significance will provide reference for follow-up research.

### 6.2 Practical implications

This paper has important practical implications for enterprise managers to promote intelligent robot services. To begin with, on technical issues, corporate technicians can establish regular technical review and optimization cycle mechanisms, improve the automatic repair capabilities of intelligent robots, and enhance rational communication between human employees and customers, thereby effectively solving the problem of intelligent robot service failures. Then, in terms of service functions, enterprise managers should establish a variety of feedback mechanisms, including user surveys, feedback channels, and discussion forums, to name a few, so that human employees can provide service guides to customers in time. Furthermore, in terms of human-robot collaboration, enterprises can reduce customer dissatisfaction with the services provided by robots through strategies such as real-time customer service and human employee intervention, effectively solve problems such as lagging business collaboration, eliminate cumbersome processes, reduce high costs, and promote the quality and efficiency of intelligent robot services, thereby further improving the service level of human-robot collaborative efforts.

### 6.3 Research limitations and future research directions

Although this paper seeks to make some contributions in theory and practice, the following limitations exist. Firstly, although the paper examines how customers perceive robots in different service failure scenarios and the impact of these perceptions on their consumption decisions, it primarily focuses on customer perspectives, neglecting the employee perspective. Therefore, future research can explore how intelligent robots impact employee behaviors in various service failure scenarios with respect to investigating the positive and negative effects of intelligent technologies on employees to bridge the gap between service failure types and employee behaviors.

Secondly, by applying Kurt Gray’s mind perception theory (Gray and Wegner, 2012), this study reorganizes the existing literature on intelligent robot service failures, forming a more unified and explicit theoretical framework to clarify customers’ psychological mechanisms in such failure scenarios and explaining the effects of service failures on customers’ cognition, attitudes, and behaviors. However, in future research on human-robot collaboration, scholars could also consider adopting other theories such as Social Judgment Theory (Roy and Naidoo, 2021), Expectancy Violation Theory (Crolic et al., 2022), Three-Factor Anthropomorphism Theory (Choi et al., 2021), and Attribution Theory (Yalcin et al., 2022) to further explore related impact mechanisms.

Thirdly, based on the types of intelligent robot service failures, this study analyzes customers’ perceptions and responses to these failures. However, the paper does not mention remedies for different service failure types of intelligent robots. Therefore, future research could explore specific remedies for service failures. For instance, robots can retrieve information from various data sources such as API interfaces, data crawling, and databases (Khder, 2021). Moreover, Ramezan et al. (2019) mentioned that human employees can introduce verification mechanisms, such as historical data comparisons and cross-validation (through multiple data sources or channels). Indeed, such service remedies related to intelligent technology will further improve the performance and interpretability of intelligent robots and effectively promote the development of AI technology to a new stage.

## 7 Conclusion

The article first sorts out and analyzes the technical service failure of intelligent robots. The discussion focused on the failure of technical components or mechanisms within the robot system, including hardware and software service failures. The discussion highlights how technical service failure leads to problems with intelligent robot service function. Then, the article discussed the functional service failure of intelligent robots (i.e., robots could not provide the required functions and services as expected or required), which includes failure of service requirements and failure of specification compliance. Similarly, the failure of an intelligent robot’s functional service will also affect the failure of its technical service. For the convenience of analysis, this article mainly divides the types of intelligent robot service failures into technical and functional service failures. Based on the above two types of service failure, this article further explored the psychological mechanism of customers in different service failure situations. After that, the article proposes corresponding adjustment mechanism from bot the robot and customer levels, mainly covering the severity of service failure, communication methods, artificial intelligent anthropomorphism, social class and interpersonal interaction needs. Finally, this article also elaborated on the impact of intelligent robot service failure on customer cognition, attitude and behavior.
